# Gracilis muscle transposition for treatment of recurrent anovaginal, rectovaginal, rectourethral, and pouch–vaginal fistulas in patients with inflammatory bowel disease

**DOI:** 10.1007/s10151-018-1918-7

**Published:** 2019-01-02

**Authors:** S. Korsun, G. Liebig-Hoerl, A. Fuerst

**Affiliations:** Department of Surgery, Caritas Clinic St. Josef, Landshuter Str. 65, 93053 Regensburg, Germany

**Keywords:** Crohn disease, Fecal incontinence, Surgical flaps, Surgical stomas

## Abstract

**Background:**

The aim of this study was to evaluate the effectiveness of gracilis muscle transposition (GMT) to treat recurrent anovaginal, rectovaginal, rectourethral, and pouch–vaginal fistulas in patients with inflammatory bowel disease (IBD).

**Methods:**

A retrospective study was conducted in patients with IBD who had GMT performed by a single surgeon between 2000 and 2018. Follow-up data regarding healing rate, complications, additional procedures, and stoma closure rate was collected.

**Results:**

A total of 30 women and 2 men had GMT. In all patients fistula was associated with Crohn's disease. In 1 female patient, contralateral gracilis transposition was required after a failed attempt at repair. The primary healing rate was 47% (15/32) and the definitive healing rate (healed by the time of data collection and after secondary procedures) was 71% (23/32). Additional surgical procedures due to fistula persistence or recurrence were performed on 17 patients (53%).At least 7 patients (21%) suffered complications including one wound infection with ischemia of the gracilis muscle. Stoma closure was successful in 18 of 31 cases of patients with stoma (58% of the patients).

**Conclusions:**

GMT for the treatment of recurrent and complex anorectal fistulas in patients with IBD patient is eventually successful in almost 2/3 of patients.

## Introduction

Treatment of recurrent and complex anorectal fistulas is challenging. Surgical therapy for fistulas in patients with inflammatory bowel disease (IBD) is associated with high recurrence rates. Fistulas in patients with IBD result from chronic relapsing inflammation with subsequent intestinal fibrosis and stricture formation, and could be triggered by the epithelial–mesenchymal transition [1–3]. The etiology of the fistula reflects the existing comorbidities, which should also be addressed intraoperatively (e.g., perineal defects and scar tissue with compromised local vascularization as a result of previous colorectal procedures). Depending on the location of the fistula, different approaches can be used, including transabdominal, transperitoneal, or local techniques. A number of local surgical methods exist, such as excision, advancement flaps, and fibrin glue or biomesh application. Autologous muscle flaps—such as the Martius, sartorius, and gracilis flaps—are muscles flaps—such as the Martius, sartorius, and gracilis flaps—are further methods for fistula closure. However, no consensus regarding the preferred method of choice has been reached. Combining different procedures after an insufficient fistula closure may be useful to improve the efficacy of a single method. Larger recent studies [4–10] on GMT, including those of Ulrich et al. [6] and Wexner et al. [9], have demonstrated some excellent results.

## Materials and methods

### Study design and patients

A two-center retrospective study was performed from January 2000 to May 2018 in patients diagnosed with IBD. All patients underwent GMT at the surgery departments of the University Hospital Regensburg and the St. Josef Hospital Regensburg, Germany, between January 2000 and May 2018 to treat anovaginal, rectovaginal (RVF), rectourethral (RUF), and pouch–vaginal fistulas. All gracilis transpositions were performed by a single surgeon (AF). IBD patients who underwent GMT strictly due to fecal incontinence and not for fistula treatment were excluded. Follow-up procedures in cases of fistula recurrence were performed by other surgeons. Prior to GMT, all patients had undergone at least one failed attempt at fistula repair using various other procedures such as fistulotomy, fistulectomy, mucosal advancement flap, fistula plug, fibrin glue application.

### Surgical technique

**Fig. 1:**
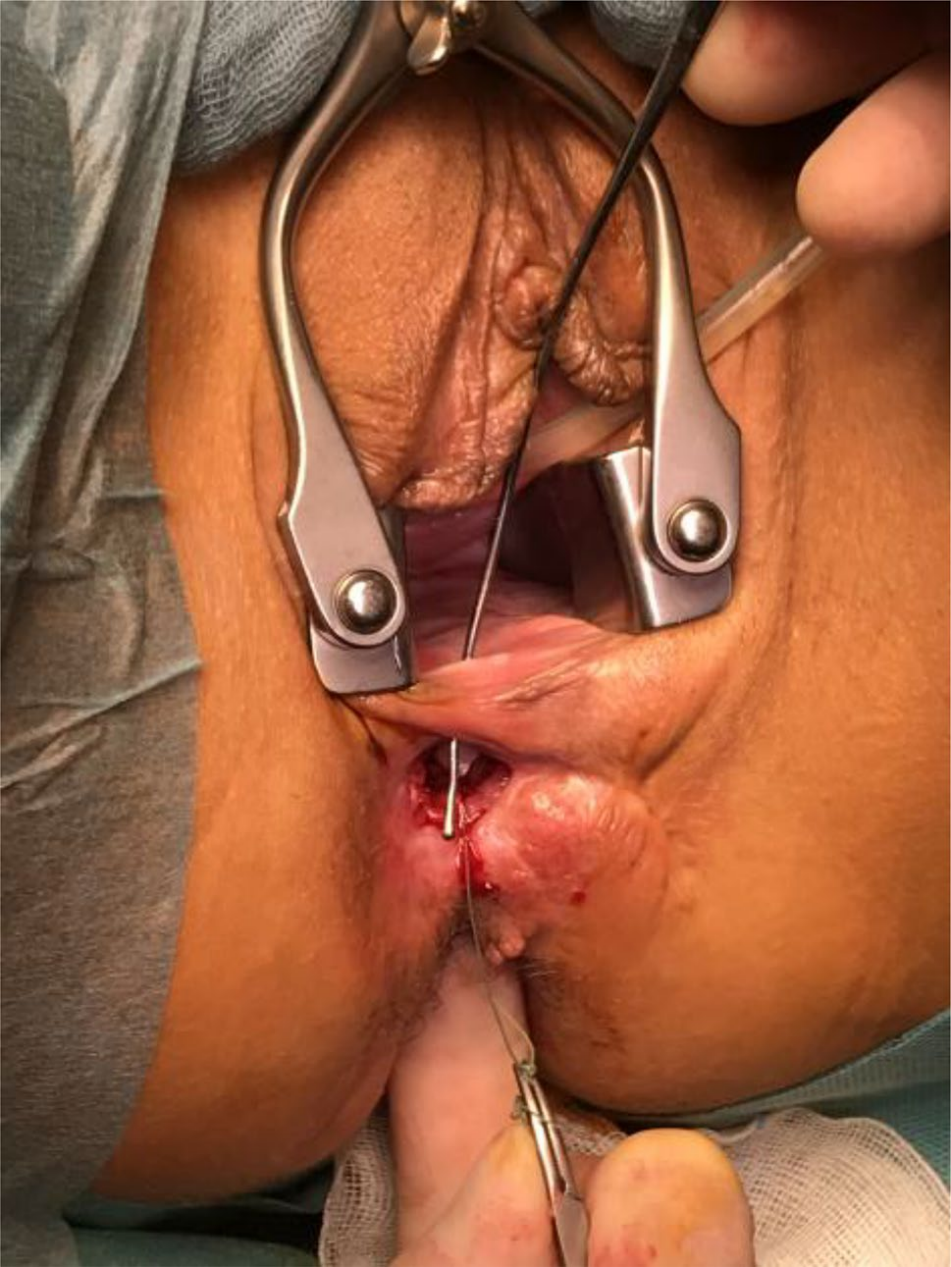
Complex rectovaginal and perineal fistula combined with a perineal defect

**Fig. 2:**
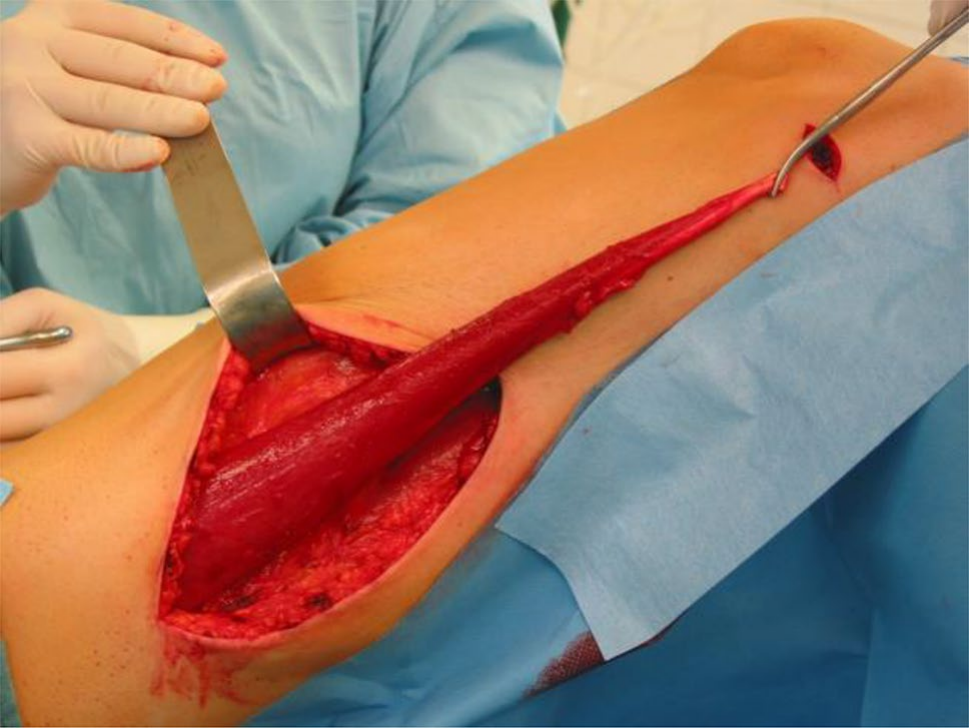
Mobilization of the gracilis muscle

**Fig. 3:**
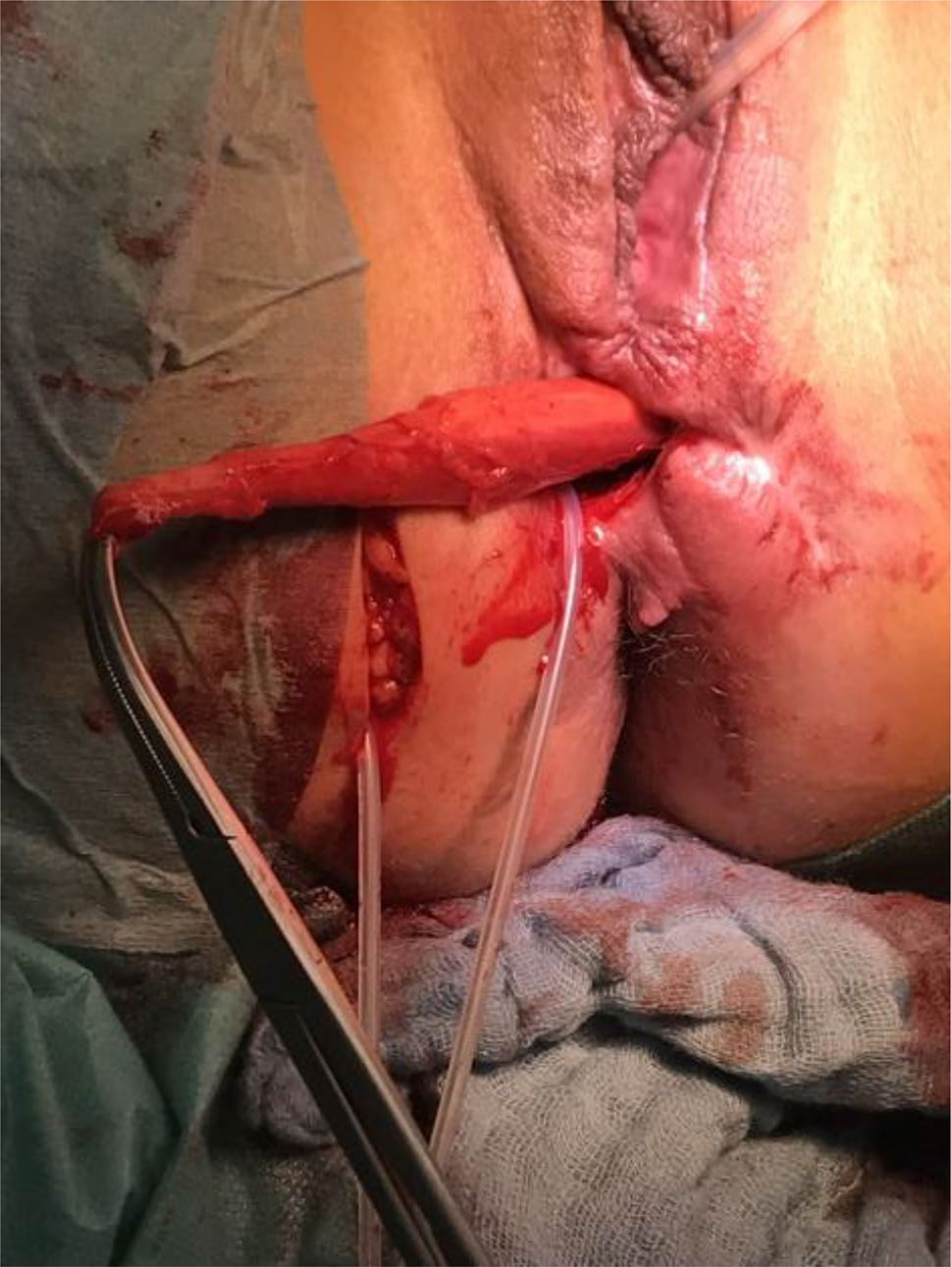
Transposition of the gracilis muscle

**Fig. 4:**
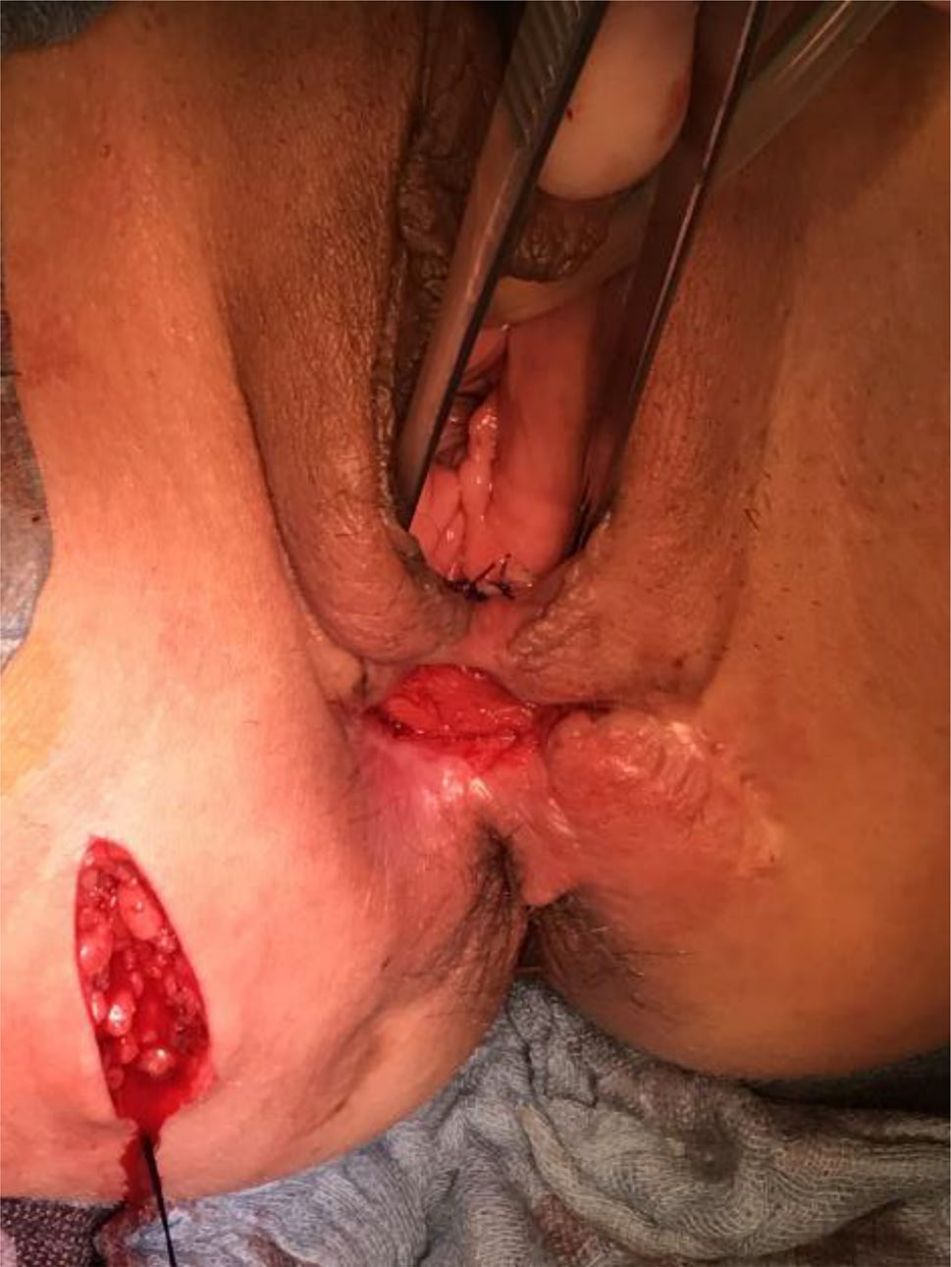
Fixation of the gracilis muscle on the contralateral pubic periost (tunneling technique)

**Fig. 5:**
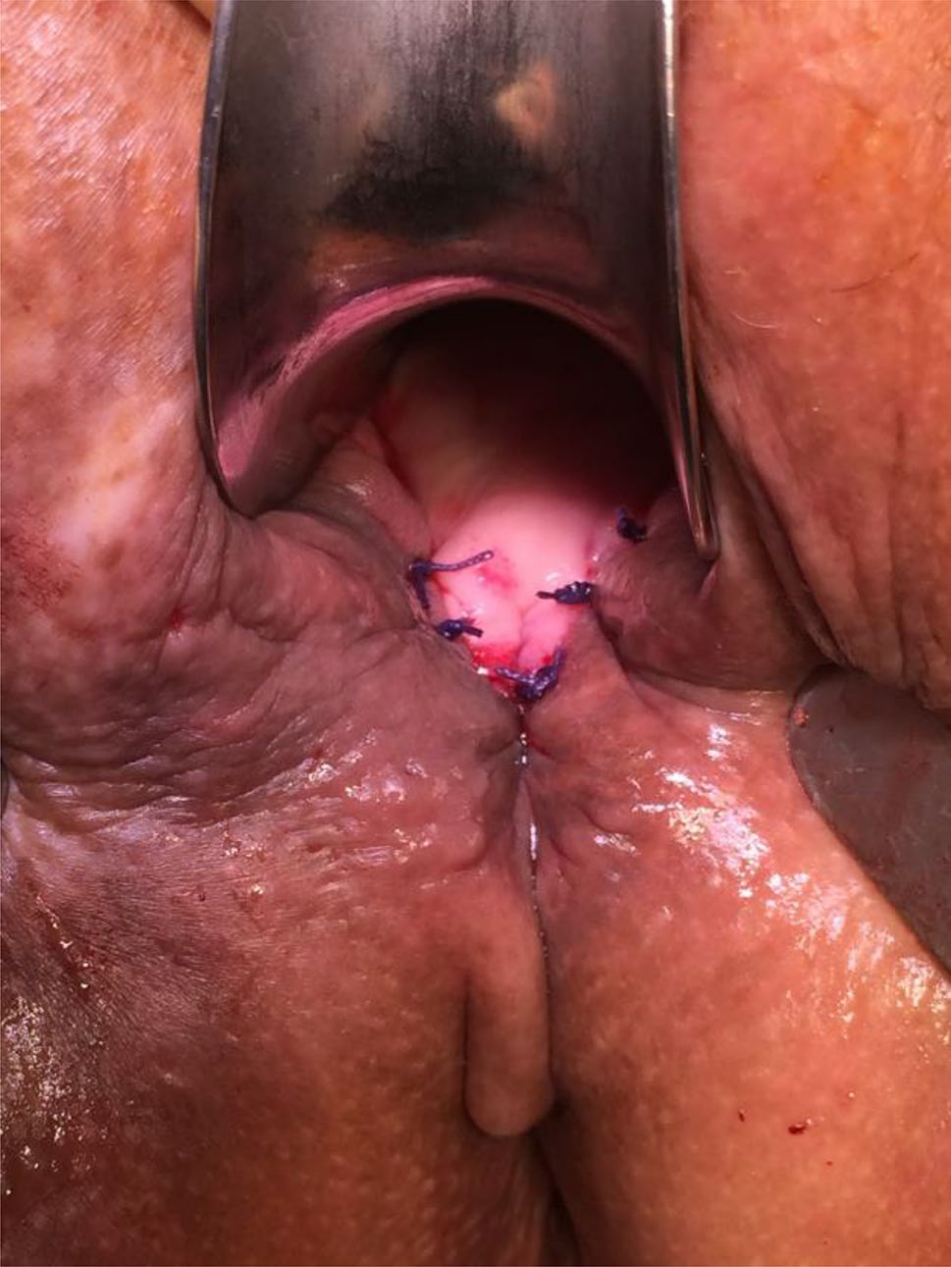
Vaginal flap to cover the gracilis muscle

**Fig. 6:**
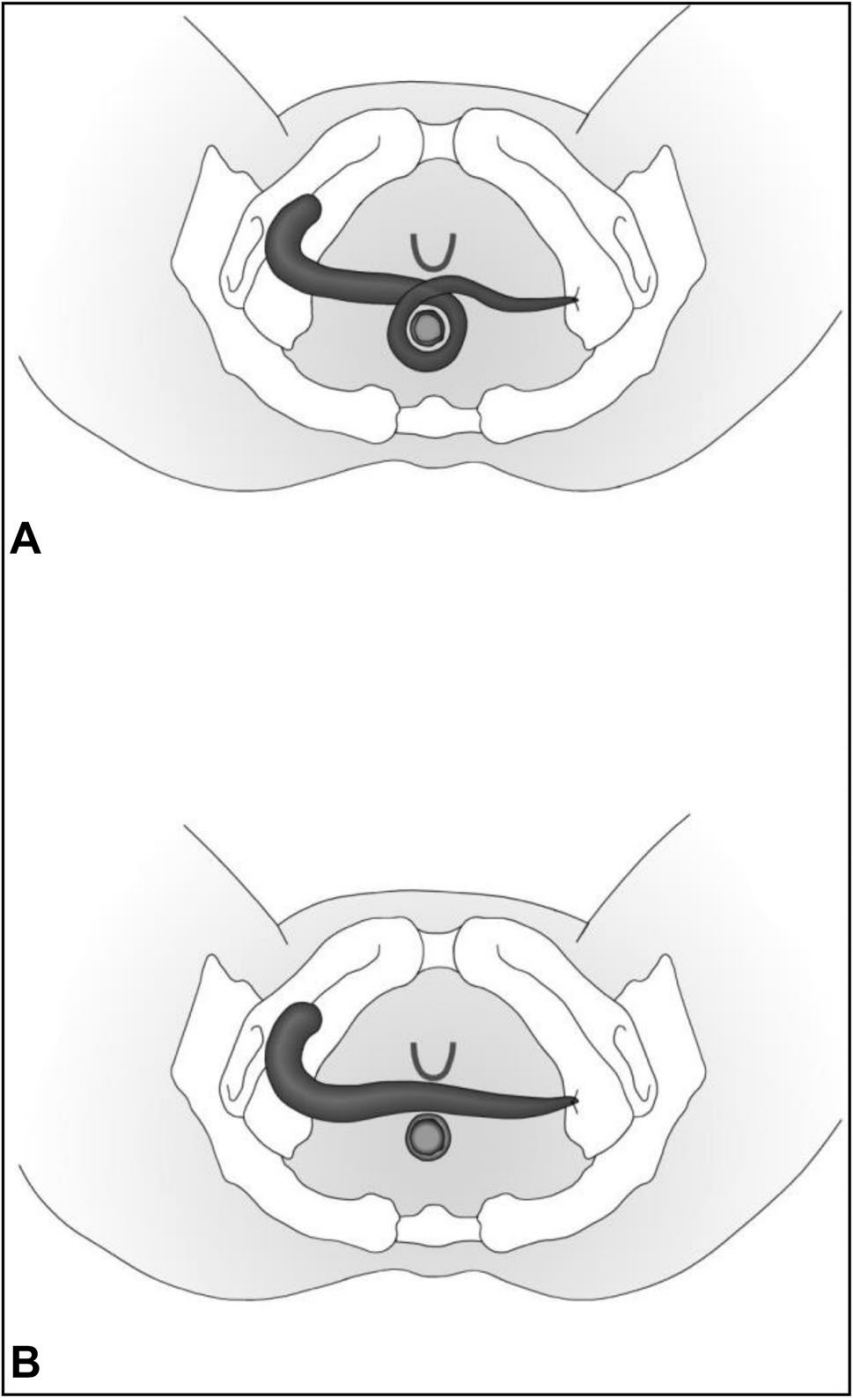
Types of gracilis muscle transposition (GMT) [10]. **a** Circular GMT, **b** transverse GMT

GMT was performed according to a standardized procedure [11]. We combined the GMT in all cases but one with a fecal diversion performing a loop ileostomy if no stoma existed. We prefer performing a GMT when a stoma is already established to reduce the local infection beforehand. Antibiotic prophylaxe with cefuroxime und metronidazole was given perioperatively. The fistula tract was identified by probing (Fig. 1) and excised completely. The defect on the rectal side was minimized with interrupted sutures. The gracilis muscle was identified and an incision measuring approximately 10–12 cm was made on the medial side of the thigh (Fig. 2). The distal muscle tendon was then identified by palpation near the pes anserinus and divided after a small incision medial to the knee joint. The muscle was mobilized and the small collateral blood vessels in the distal part of the muscle were ligated using ties for the bigger blood vessels and coagulation for the smaller ones. During mobilization, great care was taken to find a tension-free position of the neurovascular bundle on the lateral proximal side of the gracilis muscle. A subcutaneous tunnel between the ipsilateral proximal medial side of the thigh and the perineal region was created by blunt preparation. The muscle then was pulled through the tunnel and placed exactly underneath the suture line closing the rectal side of the fistula (Fig. 3), covering the rectum, placing well-vascularized tissue in the defect area (Fig. 4). Rotation of the muscle around its axis was carefully avoided. The distal tendon of the muscle was then fixed with Prolene sutures (Ethicon, Bridgewater, NJ and Cincinnati, OH, USA) to the contralateral pubic or ischial periosteum after transposition through an additional subcutaneous tunnel and a small incision on this side. Finally, the gracilis muscle was covered by a soft tissue flap (Fig. 5). If the medical history of the patient indicated fecal incontinence, the distal muscle tendon was transposed further to encircle the anal canal, and fixed at the ipsilateral pubic or ischial periosteum, thus creating a loop (gamma-loop; Fig. 6). This procedure potentially increases anal continence by contributing additional muscle tension. A secondary dynamization of the gracilis muscle remains an additional option for optimization of anal sphincter function. If no fecal diversion existed preoperatively, a protective stoma was added simultaneously. Only 1 patient opted against a protective stoma.

### Outcome assessment

The primary outcome measure was complete closure of the fistula by the first follow-up (approximately 3 months postoperatively) without additional follow-up operations. In patients with anovaginal, rectovaginal, and pouch–vaginal fistulas, the initial follow-up was performed 3 months postoperatively. A sensitive sign of the presence of a residual fistula is a feeling of air-trapping and vaginal discharge. All patients had clinical examination, including anoscopy, air insufflation, and fistula probing (in selected cases under general anesthesia). In case of uncertainty regarding a residual fistula, a water-soluble contrast enema, anorectal endosonography, or pelvic magnetic resonance imaging was performed. Stoma reversal was performed upon definitive closure of a fistula tract.

Secondary outcomes included fistula closure after follow-up procedures in cases of fistula persistence and stoma closure rate. Fistula-free time in cases of late recurrences was also documented.

### Data analysis

Information regarding patients’ demographic characteristics, number of previous operations, types of fistula, fistula closure, complications, follow-up procedures, and stoma closure was collected retrospectively from medical records. Data were gathered in a Microsoft Excel Sheet Version 2010 (Microsoft Corporation, Redmond, WA, USA). Data are presented using summary statistics in the form of means and median values for quantitative data and percentages for qualitative data.

### Ethical considerations

Due to the retrospective nature of the study, formal consent was not required. The study was approved by the local ethics committee of the authors’ study center.

## Results

A total of 32 patients (2 male, 30 female; mean age 39 years, range 24–55 years) with IBD underwent gracilis transposition or re-transposition for a recurrent fistula at the authors’ institutions between January 2000 and March 2018 and attended at least the first follow-up visit at 3 months. Two patients with less than 3 months of follow-up were also included (one because of graft ischemia and the other because of a persistent fistula) to minimize selection bias and present a more realistic recurrence and complication rate. All patients had undergone diverse previous surgical procedures for fistula closure (mean 2 procedures, range 1–25 procedures) before the gracilis transposition. Mean follow-up time after GMT was 47 months (range 1–144 months). After GMT, some patients suffered a recurrence or had a smaller residual fistula, prompting different follow-up procedures to achieve definitive closure (Tables 1, 2 and 3).

**Table 1:** Additional procedures after GMT for patients with recurrence

Patient	Date of GMT	Follow-up procedures	Last follow-up
I	09/2002	12/2002 stoma closure 12/2005 recurrence 03/2006 abscess excision and fistulectomy 05/2006 fistulectomy and double barrel ileostomy	09/2013 fistula not closed
II	04/2005	07/2005 stoma closure 02/2010 recurrence/ new fistula 02/2010 fistulectomy 04/2010 fistulectomy, rectal muscle flap and fistula plug 08/2010 vaginal advancement flap 09/2010 loop sigmoidostomy 11/2010 fistulectomy and vaginal flap 03/2011 stoma closure	03/2011 fistula closed
III	04/2013	07/2013 stoma closure 10/2013 recurrence 11/2013 biomesh 12/2013 seton 06/2015 vaginal flap 03/2018 seton	03/2018 fistula not closed
IV	06/2009	09/2009 stoma closure 08/2014 recurrence after crohn relapse 10/2014 rectal flap 11/2014 fistulectomy and rectal flap 02/2015 rectal flap and double barrel colostomy 03/2015 rectal and vaginal flap 05/2015 rectal flap 09/2017 fistulectomy and rectal flap 02/2018 martius flap	03/2018 fistula not closed
V	12/2015	02/2016 stoma closure 04/2017 abscess excision 07/2017 fistulectomy	08/2017 fistula closed
VI	02/2016	09/2016 stoma closure 11/2016 fistulectomy	12/2017 fistula closed
VII	10/2005	07/2006 stoma closure 03/2007 recurrence 03/2007 abscess excision 12/2007 seton and double barrel ileostomy	12/2007 house ban, fistula not closed
VIII	05/2016	12/2016 recurrence 12/2016 abscess excision 02/2017 fistulectomy and rectal flap 03/2017 abscess excision 03/2018 stoma closure	03/2018 fistula closed
IX	05/2006	10/2006 recurrence 11/2006 fistulectomy and rectal advancement flap	11/2009 fistula closed

**Table 2:** Additional procedures after GMT for patients with fistula persistence

Patient	Date of GMT	Follow-up procedures	Last follow-up
I	03/2016	Persistence 05/2016 fistulectomy 04/2017 fistulectomy and rectal flap 03/2018 fistulectomy and rectal flap	03/2018 fistula not closed
II	05/2014	Persistence 04/2015 rectal flap 11/2016 stoma closure	11/2016 fistula closed
III	03/2015	Persistence 06/2015 rectal flap 10/2015 stoma closure	10/2015 fistula closed
IV	01/2000	Persistence 03/2000 vaginal flap 09/2007 proctocolectomy and converting loop ileostomy in double barrel ileostomy (not because of fistula) 05/2010 abscess excision	01/2012 fistula closed
V	07/2014	Persistence 09/2014 vaginal flap 02/2015 rectal flap 05/2015 vaginal flap 12/2015 vaginal flap 03/2017 seton	11/2017 fistula not closed
VI	02/2016	Persistence 04/2016 vaginal flap	04/2016 fistula not closed
VII	05/2012	Persistence 10/2012 fistulectomy, vaginal and rectal flap and fistula plug 02/2013 fistulectomy and vaginal flap	04/2013 fistula not closed
VIII	07/2015	08/2015 wound infection with necrosis and excision of the gracilis flap	10/2015 fistula not closed

**Table 3:** Fistula closure rate after follow-up procedures depending on gender

Female IBD patients	Male IBD patients
*n* = 21/30	*n* = 2/2
70%	100%

*IBD* inflammatory bowel disease

Of the 32 patients with IBD, 1 developed a wound infection with necrosis of the gracilis flap and 7 had a residual fistula at the first postoperative follow-up and required additional procedures. During the subsequent follow-up period, 9 further patients had a recurrent or new fistula and one patient had an abscess without a verification of a fistula (median time after GMT among these 9 patients before recurrence: 17 months; range 5–62 months). In 7 of those cases a recurrence took place after stoma closure (median time after stoma closure until recurrence 17 months, range 2–59). It was difficult to distinguish whether the patient had a recurrence or a new spontaneous fistula due to Crohn’s disease (CD). In 1 patient, a recurrence was documented directly after a CD relapse. The primary fistula closing rate where the fistula stayed closed during the follow-up and no further procedures were needed was 47% (15/32).

In total, an additional 43 operations were required for the 17 patients with recurrences and persistence of a fistula after GMT to achieve a definitive fistula closure rate after recurrences of 71% (23/32) among all 32 patients (Table 1). Of these operations, 17 were performed to close a recurrent or persistent fistula in 8 patients (2.1 follow-up procedures per patient per closed fistula after recurrence or persistence). Despite performing a total of 26 other follow-up procedures on 9 other patients, those 9 other patients had a persistent fistula and in one another case there is a suspicion of a recurrence due to an abscess without a clinical proof of a fistula.

At the time of GMT, 15 patients were not taking any specific CD medication (Table 4). In six of those cases the fistula healed without follow procedures. Only in 4 of those 15 patients (healing rate 73%) without specific CD medication at the time of GMT a fistula remained after follow procedures. In 5 of the 17 cases (healing rate 71%) where patients took a specific CD medication a residual fistula remained after follow procedures. However, the p value for comparing the healing rate with or without specific CD medication is 0.589 and thus not of statistical significance.

**Table 4:** CD medication at the time of GMT

Patient no.	Gender	CD onset (year)	GMT (year)	Fistula closed	Perioperative IBD medication	IBD Medication before GMT	IBD medication after GMT
1	f	1984	2000	y	Short chain fatty acid enema	1997 steroids; 1998 azathioprine	2002 and 2005 mesalazine foam, steroids
2	f	1985	2001	y	Azathioprine, steroids, colifoam	Unknown	Unknown
3	f	1989	2004	y	None	Unknown	Unknown
4	f	1988	2004	y	Azathioprine and steroids	2001 azathioprine, steroids	2007 azathioprine, steroids
5	f	1984	2010	y	None	Unknown	Unknown
6	f	2003	2010	y	Mercaptopurine	02/2010 adalimumab, mercaptopurine	Unknown
7	m	2000	2005	y	None	Unknown	09/2005 azathioprine
8	m	1989	2012	y	Azathioprine	11/2011 azathioprine	12/2012 azathioprine
9	f	2002	2014	y	adalimumab (pause)	12/2013 adalimumab	03/2015 adalimumab
10	f	1994	2010	y	Adalimumab, azathioprine, steroids	Unknown	Unknown
11	f	2006	2005/2006	y	None	Unknown	Unknown
12	f	1988	2002	n	Azathioprine, steroids, mesalazine foam	Unknown	2005, 2006, 2013 azathioprine
13	f	2001	2005	y	None	Unknown	2010 none; 2011 none; 2014 steroids
14	f	1984	2006	y	None	Unknown	2016 azathioprine
15	f	1997	2005	n	None	Infliximab	2007 azathioprine, steroids
16	f	2012	2012	n	None	None	None
17	f	1995	2012	n	Steroids	Unknown	Unknown
18	f	2005	2013	n	None	Unknown	07/2013 sulfasalazine supp
19	f	2007	2009	n	Methotrexate (MTX)	Unknown	09/2009 MTX; 2014–2015 MTX, steroids; 10/2015–2016 steroids, golimumab; 09/2016 adalimumab
20	f	1999	2014	y	Sulfasalazine	02/2012 sulfasalazine	2014 sulfasalazine; 2015 sulfasalazine, steroids, azathioprine
21	f	2002	2014	y	None	Unknown	11/2016 none
22	f	2006	2015	y	None	10/2014 steroids, adalimumab	10/2015 vedolizumab
23	f	1988	2000	y	Azathioprine, steroids, sulfasalazine	Unknown	10/2002 steroids, azathioprine; 2006 azathioprine
24	f	2010	2015	y	Azathioprine	Azathioprine, mesalazine	10/2015 azathioprine
25	f	2005	2016	y	None	Unknown	None
26	f	1998	2015	y	None	Unknown	None
27	f	1995	2015	y	Sulfasalazine	Unknown	07/2017 steroid foam
28	f	2005	2016	y	None	Infliximab	Unknown
29	f	2007	2016	y	Vedolizumab (pause)	Vedolizumab	02/2017 vedolizumab; 03/2018 ustekinumab
30	f	1992	2015	n	None	Unknown	None
31	f	1998	2016	n	Mercaptopurine	Mercaptopurine	2016 mercaptopurine
32	f	2008	2016	n	Azathioprine, steroids	Azathioprine, steroids	05/2016 azathioprine, steroids; 04/2017 azathioprine

*f* female, *m* male, *y* yes, *n* no, *GMT* gracilis muscle transposition, *CD* Crohn’s disease, *IBD* inflammatory bowel disease

GMT was performed without fecal diversion in 1 patient and the fistula healed anyway. In 19 patients, the stoma could be reversed after fistula closure and in 2 patients, stoma reversal is intended after fistula healing. A late recurrence after stoma reversal was suffered by 7 patients, 4 of whom received a new stoma with follow-up surgical procedures thereafter. As a result, in 3 of the 7 latter cases, fistula closure was achieved. In summary, a stoma reversal after follow-up procedures due to fistula persistence or recurrence was achieved in 18 of 31 (58%) of the stoma patients and in two other cases where a fistula closure has been achieved a stoma closure is being planned.

Mean age was 42 years (median 43 years, range 24–54 years, 95% CI 33–51 years) in the patients with complete remission after GMT and 40 years (median 41 years, range 27–55 years, 95% CI 33–49 years) in the patients who suffered a recurrence (*p* = ns).

The mean number of operations before GMT was 4.3 (median3, range 1–20) and 4.4 (median2, range 1–25) in the groups who did not and did suffer a recurrence, respectively (*p* = ns).

There was no difference in fistula closure rates between patients who received a gamma-loop of gracilis muscle to the ipsilateral pubic ramus (7/10, 70%) and those who underwent GMT on the contralateral pubic ramus (15/22, 68,1%) (*p* = ns).

Data were collected on fistula closure and the closure rate by fistula etiology was calculated (Table 5).

**Table 5:** Fistula closure rate among patients with IBD

Fistula types	Fistula closure rate	Stoma closure rate
Rectovaginal fistula *n* = 21	71% (incl. one patient with an abscess after GMT without fistula proof)	55% (1 patient operated without stoma and 1 patient opting against stoma closure after fistula closure)
Anovaginal fistula *n* = 2	50%	50%
Anal fistula *n* = 4	100%	100%
Pouch fistula *n* = 3	67%	67%
Complex fistula *n* = 1	0%	0%
Rectourethral fistula *n* = 1	100%	0%^a^
Total *n* = 32	71%	58%

*IBD* inflammatory bowel disease

^a^Stoma closure being planned

### Complications

Aside from 1 case of wound infection with ischemia of the gracilis muscle, 1 case of postoperative hemorrhage in the thigh 2 cases of suture granuloma and complaints about scar tissue and some numbness on the operated thigh, no other major complications following harvesting and inserting of the gracilis muscle were documented.

When a new fecal diversion was performed during the same session, some patients needed postoperative intravenous fluid and laxative agents due to ongoing paralytic ileus, but no surgical interventions were needed (Table 6).

**Table 6:** Complications

Complications	Number of patients
Postoperative hemorrhage	1
Thigh swelling	1
Lymphoedema	1
Suture granuloma	2
Knee pain	1
Thigh hypoesthesia	2
Necrosis of the gracilis muscle	1

## Discussion

Muscle grafts, particularly gracilis muscle grafts, have been in use since the 1930s. It should be noted that the gracilis muscle does not close the existing fistulas per se, but is used to reduce dead space in the perineum and improve vascularization and healing in areas with compromised blood supply due to soft tissue and muscle loss as a result of previous operations. GMT can be combined with other surgical interventions and, thus, improve the rate of fistula closure.

Alternative approaches include transperineal, endorectal, endovaginal, and transabdominal procedures. Fistula closure can be achieved directly using sutures or glue, with a mucosal flap or biomaterials, via partial bowel resection with anastomosis, or through muscle flap transposition. Fecal diversion can be performed as a stand-alone treatment or used perioperatively as a supplementary measure in some surgical procedures.

Biomaterials represent a relatively new method of treating fistulas. Benefits include less intraoperative trauma and a decreased impact on fecal continence, while treating trans-sphincteric fistulas with moderate–good outcomes [12–17]. Chan et al. [18] showed good results using a fistula plug in simple fistulas, but also a decrease in efficacy after multiple procedures. Other studies showed a high rate of postoperative sepsis after implantation of fistula plugs alone [19, 20]. In combination with a draining seton or an advancement flap, the success rate increases. Long-term studies of this method are necessary to judge its efficacy.

Panés et al. [21–23] describe a promising new therapeutic approach to close treatment-refractory complex perianal fistulas in CD using a single intralesional injection of allogeneic expanded adipose-derived stem cells. This method achieved a 50% remission rate (53 of 107 patients) in an intention-to-treat population. The most common treatment-related adverse events were anal abscess and proctalgia.

The fistula closing rates by sex are shown in (Table 3); however, the significantly smaller number of patients in the male group makes comparisons difficult. All in all, we achieved a definitive fistula closing rate of 71%. Our study including 32 patients is the largest study of performing GMT on IBD patients in the literature so far.

Compared to other studies on GMT, the current study group consisted entirely of patients with IBD, which is generally associated with lower fistula healing rates using different procedures [6, 9, 24–26]. Some patients with IBD show apparent late recurrences; however, some of these may be attributable to spontaneous development of new fistula due to CD activity, rather than a recurrence of a previous fistula.

No significant difference in terms of age was detected between the groups with remission and recurrence. Therefore, the authors would recommend the procedure for IBD patients of all ages. There was also no significant difference in healing rate between patients taking CD specific medications and patients not taking any CD specific medications at time of GMT. 

Fistula persistence after GMT was detected no later than at the first follow-up after GMT, and was treated accordingly. Late recurrences, however, appeared unequally distributed after a mean of 24 months after GMT (median 23 months, range 5–62 months). Almost half of the recurrences took place 5–7 months after GMT and the others appeared 1.5–5 years postoperatively. Another follow-up with clinical examination and anorectal sonography 6–9 months after GMT might be advantageous for earlier detection of a recurrence and initiation of treatment.

Adequate treatment of IBD seems to have a positive impact in preventing late recurrences. Due to the small number of patients in the present cohort, it was not possible to detect whether success after GMT correlated with the different types of CD medication.

The fact that all GMTs were performed by a single surgeon using a standardized procedure reduced the possibility of the outcome being influenced by varying skill levels of different surgeons.

The study has some limitations. First, only a small number of patients were included. Further studies with larger patient numbers are needed. There are, however, only a few similar studies on fistula treatment in IBD patients in the recent literature.

Due to its retrospective design, the current study lacks a uniform follow-up period. A defined interval between the procedure and examination of the patients could improve comparison between the different patients and success rates. The follow-up at 3 months postoperatively did not apply to all patients, mainly because some suffered a persistent fistula and were examined earlier than after 3 months. The next follow-up after the 3-month follow-up was also somewhat heterogeneous, since most patients received a further follow-up only due to complaints; a large proportion of the patients who achieved complete remission and stoma closure did not attend further examinations.

Another critical point of the study is the correlation between GMT and the fistula closure rate when additional procedures were needed. It is impossible to determine the exact extent of the contribution of GMT to the fistula closure rate when additional operations were performed after GMT. The authors are convinced that the gracilis muscle is the basis for the success of additional fistula operations. Larger trials directly comparing GMT to other surgical procedures with a long follow-up period should be performed to better understand its benefits. This would contribute to determining the best way to treat recurrent fistulas in patients with IBD (Table 7).

**Table 7:** Comparison of pre-existing studies

Author	Year	Number of patients	Fistula type	Follow-up period (months)	Success rate
Zmora et al. [8]	2003	11	RUF	4–40 (median 18, 2)	100%
Fürst et al. [10]	2008	12	RVF and anovaginal	Mean 40	91.6%
Wexner et al. [9]	2008	17	15 × RVF 2 × pouch–vaginal		33% (Crohn’s) and 75% (non-Crohn’s)
Wexner et al. [9]	2008	36	RUF		97%
Lefevre et al. [5]	2009	8	RVF	4–55 (median 28)	88%
Ulrich et al. [6]	2009	35	9 × RVF 26 × RUF	Mean 28 ± 15	94%
Maeda et al. [7]	2011	14	Complex fistula	1–88 (median 10)	64%
Maeda et al. [7]	2011	4	Persistent nonhealing perineal sinus	1–88 (median 10)	50%
Chen et al. [4]	2013	19	RUF and RVF	6–35 (median 18)	94.7%
Current study	2018	32	RVF, RUF, pouch–vaginal, and anovaginal	1–144 (mean 47)	71%

*RUF* rectourethral fistula, *RVF* rectovaginal fistula

## Conclusions

Our results are promising and suggest that GMT is a safe and very effective option for the treatment of recurrent fistulas in IBD patients. It should be noted that for complete fistula closure, follow-up procedures may be necessary. The option of dynamization of the muscle creates the possibility of improving fecal continence and, thus, quality of life. The authors suggest that in cases of recurrence after initially addressing a fistula with conventional methods, and after repeated unsuccessful procedures, GMT should be considered.
